# Strengthening pharmacovigilance through patient reporting: a scoping review of awareness, barriers, and facilitators

**DOI:** 10.1080/20523211.2026.2651405

**Published:** 2026-04-13

**Authors:** Adeel Aslam, Hafizah Abdul Majid, Nimra Muhammad Boota, Asma Ghulam Mustafa, Sumera Saeed Akhtar

**Affiliations:** a Faculty of Pharmacy, Department of Pharmacy Practice and Clinical Pharmacy, University Teknologi MARA, Puncak Alam, Malaysia; b Department of Pharmacy and Biomedical Sciences, MAHSA University, Jenjarom, Malaysia; c Department of Pharmacy, University of Lahore, Punjab, Pakistan; d Faculty of Pharmacy, University of Lahore, Punjab, Pakistan; e Department of Primary Health Care, Faculty of Medicine, University of Otago, Dunedin, New Zealand

**Keywords:** Adverse drug reactions, patient engagement, pharmacovigilance, scoping review, reporting behaviour

## Abstract

**Background:**

Adverse drug reactions (ADRs) significantly impact global healthcare systems, yet patient participation in pharmacovigilance remains underutilised. This scoping review synthesises evidence on patient awareness, barriers, and facilitators for ADR reporting to inform strategies for enhancing pharmacovigilance systems.

**Methods:**

Following the Arksey and O’Malley framework, we conducted a scoping review of studies published up to 2025 across PubMed, Scopus, Embase, Web of Science, Cochrane Library, and Google Scholar. Two independent reviewers screened titles, abstracts, and full texts, extracted data, and synthesised findings using thematic and descriptive analyses.

**Results:**

A total of 1,420 articles were identified, of which 42 studies from 25 countries met the inclusion criteria. Most employed quantitative descriptive designs (n = 27), followed by mixed-methods (n = 7) and qualitative approaches (n = 5). Sample sizes ranged from 15 participants to over 500,000 ADR reports from national databases. Data collection methods included questionnaires, interviews, and electronic surveys, while six studies analysed secondary data from pharmacovigilance systems such as VigiBase and the Yellow Card Scheme. Key barriers included limited knowledge, complex reporting processes, and a lack of feedback, whereas facilitators included healthcare professional support and simplified, user-friendly platforms.

**Conclusions:**

Patient reporting is hindered by systemic and educational gaps, especially in LMICs. Strategic interventions, such as simplified reporting mechanisms, nationwide awareness campaigns, digital tool integration, and healthcare provider training, are critical to empower patients as active pharmacovigilance contributors. Future efforts must prioritise culturally tailored approaches and equitable access to reporting infrastructure.

## Background

Adverse drug reactions (ADRs) impose a significant and persistent burden on healthcare systems globally, contributing to substantial patient morbidity, mortality, and escalating costs (Kommu et al., 2025). Approximately 6.5% of hospital admissions are attributable to drug-related issues, and ADRs occur in an estimated 10–20% of hospitalised patients, underscoring a persistent and significant challenge to patient safety and healthcare systems (Davies et al., 2010). This burden is further intensified by demographic shifts towards older populations experiencing polypharmacy and the accelerated introduction of novel therapeutics with potentially unknown long-term safety profiles (Alomar, 2014). Pharmacovigilance (PV) systems play a vital role in reducing such risks and are defined as the science and activities involved in detecting, assessing, understanding, and preventing adverse effects or any other problems related to medicines (Avery et al., 2011). The efficacy of PV fundamentally relies on robust ADR reporting to generate data for signal detection and risk management (Rolfes et al., 2017).

Traditionally, ADR reporting depend solely on healthcare professionals (HCPs). However, there is an increasing recognition of the value of direct patient reporting of adverse drug reactions as a crucial source of pharmacovigilance data (Inácio et al., 2017). Patient-submitted reports provide distinctive insights by delivering comprehensive descriptions of symptoms, outlining effects on everyday activities, and offering precise timelines, thereby capturing perspectives and experiences that are frequently absent from healthcare professional documentation (Bagheri, 2024). Patient involvement enhances signal detection, particularly for rare, delayed-onset, or quality-of-life-impacting ADRs, contributing significantly to a more comprehensive safety profile of medicines (Aung et al., 2023). Despite their acknowledged value, patient under-reporting remains a significant global challenge to effective pharmacovigilance, largely driven by persistently low public awareness of national ADR reporting systems (van Hunsel et al., 2019). This is reflected regionally, where in Malaysia, only 8% of the public are aware of the national ADR reporting system [10], while in Saudi Arabia, just 15.7% of participants report familiarity with pharmacovigilance (Almubark et al., 2020).

Patients frequently cite barriers including uncertainty about attributing symptoms to a drug, perceptions of overly complex reporting procedures, and frustration due to a lack of feedback following report submission (Inácio et al., 2017; van Hunsel et al., 2019). Moreover, global efforts focus on improving accessibility, such as the US FDA MedWatch programme, which provides multiple accessible channels for consumer reporting (Fang et al., 2014). Similarly, the UK Yellow Card Scheme has innovated with digital tools like a mobile application to lower reporting barriers (Shalviri et al., 2024). In many countries, the collection and evaluation of adverse drug reaction (ADR) reports from healthcare professionals, pharmaceutical companies, and the public are coordinated by national pharmacovigilance authorities (Khan et al., 2023; Valinciute-Jankauskiene et al., 2021). However, direct patient contributions to ADR databases often remain disproportionately low, reflecting a significant gap in public engagement and highlighting the need for targeted interventions (Shafei et al., 2023). To enhance patient reporting, effective strategies should include nationwide public awareness campaigns, simplification of the reporting process through user-friendly digital platforms, and the establishment of systematic feedback mechanisms to acknowledge and inform reporters (Hariraj & Aziz, 2018). Strengthening patient involvement is critical for robust drug safety monitoring. Patient reports provide distinctive real-life experience data, offering in-depth details about ADR manifestations and consequences often absent from HCP reports (Rolfes et al., 2015). Evidence shows that patient-reported ADRs often contain information not present in healthcare professional submissions, making them invaluable for identifying rare, delayed, or previously undocumented reactions (Aung et al., 2023). Therefore, this scoping review aims to map and critically appraise the existing literature on patient awareness, participation, barriers, and facilitators of ADR reporting. By consolidating evidence from diverse healthcare systems and populations, the review seeks to identify key challenges and opportunities to strengthen patient-centred pharmacovigilance and guide future interventions.

## Methods

**Figure 1.**
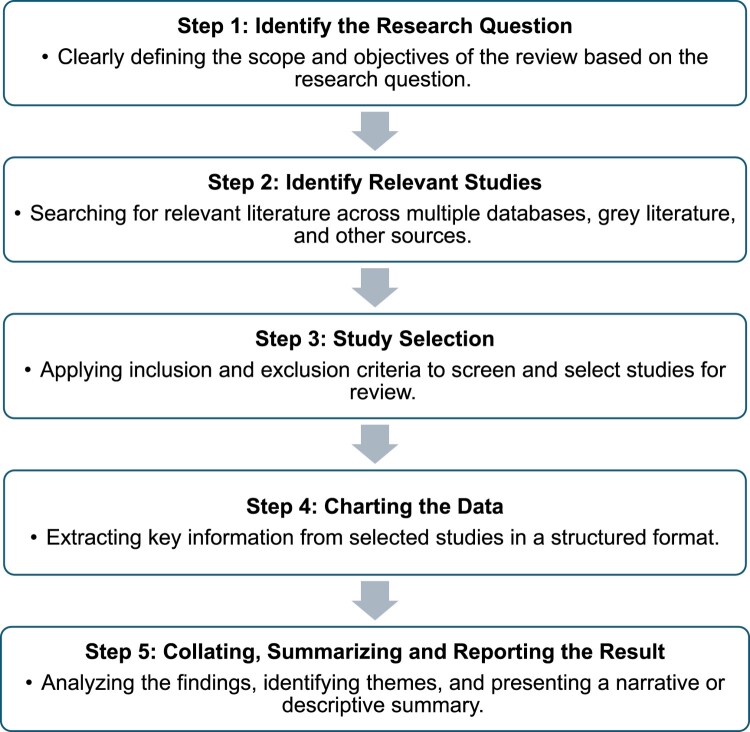
Framework for conducting a scoping review based on Arksey and O’Malley.

This scoping review was conducted in accordance with the Preferred Reporting Items for Systematic Reviews and Meta-Analyses extension for Scoping Reviews (PRISMA-ScR) guidelines. The methodological approach followed Arksey and O’Malley’s five-stage framework:

1. Identifying the research question (structured using the PCC framework),
2. Identifying relevant studies,
3. Selecting studies,
4. Charting the data, and
5. Collating, summarising, and reporting results (Figure 1).

## Research question (PCC framework)

The central research question was guided by the PCC (Population-Concept-Context) framework to ensure a comprehensive exploration of the literature:

- Population: Patients
- Concept: Awareness, understanding, barriers, motivators, and sociodemographic factors influencing participation
- Context: Pharmacovigilance (ADR reporting) across various healthcare settings

**Refined Question:**

What is known in the existing literature about [P – patients’] [C – awareness, understanding, barriers, and motivators] regarding [C – adverse drug reaction (ADR) reporting in pharmacovigilance systems across different healthcare settings]?

## Study selection & data synthesis

To address this question, a systematic search was conducted, followed by screening and data extraction. A narrative synthesis was applied to categorise studies by:

- Research designs including qualitative, quantitative, mixed methods,
- Reported barriers and motivators,
- Levels of patient awareness, and
- Sociodemographic influence.

## Search strategy

A comprehensive search strategy was implemented across PubMed, Scopus, Embase, Web of Science, Cochrane Library, and Google Scholar. The initial search was conducted in October 2024 and updated in August 2025 to ensure inclusion of the most recent evidence. Search terms combined relevant keywords with controlled vocabulary (e.g. MeSH and Emtree terms) related to patient awareness, attitudes, practices, and behaviours in ADR reporting, with Boolean operators applied to refine results. The complete PubMed search strategy was developed, and equivalent strategies were adapted for Scopus, Embase, Web of Science, the Cochrane Library, and Google Scholar to accommodate database-specific syntax and indexing. Grey literature was identified through targeted searches of regulatory agency websites (e.g. WHO, EMA, FDA) and performing manual reference list screening of included studies. For efficient data handling, all search results were imported into EndNote (version 21) for reference management, and duplicate records were removed using Microsoft Excel before commencing the screening process to ensure data accuracy.

## Eligibility criteria

To ensure the inclusion of relevant evidence, studies were considered eligible if they examined patient awareness, involvement, or experiences related to ADR reporting, employed primary research designs such as qualitative, quantitative, or mixed methods, and were published up to August 2025. In contrast, studies were excluded if their focus was limited to healthcare professionals without incorporating patient data, presented non-original research such as reviews, editorials, or commentaries, or were available only as abstracts or conference proceedings.

## Data extraction and quality assessment

Data were extracted using a standardised form capturing key study characteristics, namely author (year), country, study aim, sample size, study design, data collection methods, instruments, setting, results, conclusions, identified barriers, strategies, and the reported level of awareness and involvement. These details are summarised in Table 1, which provides a comprehensive overview of all included studies. Then, the extracted data items (e.g. awareness rates, reporting behaviours, barriers, facilitators, and sociodemographic factors) were mapped to the ‘Concept’ element of the PCC framework to ensure alignment with the research question. Furthermore, studies were categorised into three awareness and involvement levels based on predefined criteria to facilitate comparative analysis:

**Table 1.** Criteria for categorising levels of awareness and reporting rates in ADR reporting.

Level	Awareness (%)	Reporting rate (%)
Low	<20	<15
Moderate	20–50	15–40
High	> 50	> 40

Awareness refers to the proportion of patients knowledgeable about ADR reporting, while the reporting rate reflects the percentage of patients who have submitted an ADR report. Categorisation criteria were predefined to enable consistent classification of included studies. To minimise potential bias, title and abstract screening were performed independently by two reviewers, followed by a full-text assessment. Any disagreements were resolved through discussion with a third reviewer consulted when required. The quality of the included studies was evaluated using the 2018 version of the Mixed Methods Appraisal Tool (MMAT), which is suitable for qualitative, quantitative, and mixed-method research designs (Supplemental Material).

## Data synthesis

To summarise the overall findings, descriptive statistics were employed to present study characteristics such as geographical distribution, methodology, and sample size. In parallel, qualitative findings were analysed thematically following Braun and Clarke’s framework. This process enabled the identification of key themes across studies, including levels of patient awareness, common barriers and motivators for ADR reporting, the influence of sociodemographic factors, and existing strategies to improve engagement. Extracted data items such as the awareness rates, reporting behaviours, barriers, facilitators, and sociodemographic factors, were mapped to the ‘Concept’ element of the PCC framework to ensure alignment with the research question. Given the qualitative nature of much of the extracted data, NVivo software facilitated systematic coding and thematic analysis, supporting consistency and transparency in theme identification. A narrative synthesis approach was then applied, organising findings around the review’s core objectives to ensure a coherent exploration of the evidence landscape. The studies included in the review encompassed diverse designs, including qualitative interviews, cross-sectional surveys, and retrospective analyses of databases, and differed in their target populations and outcome measures. This heterogeneity was addressed through narrative synthesis and thematic categorisation, rather than statistical pooling, to ensure meaningful integration of findings.

## Results

**Figure 2.**
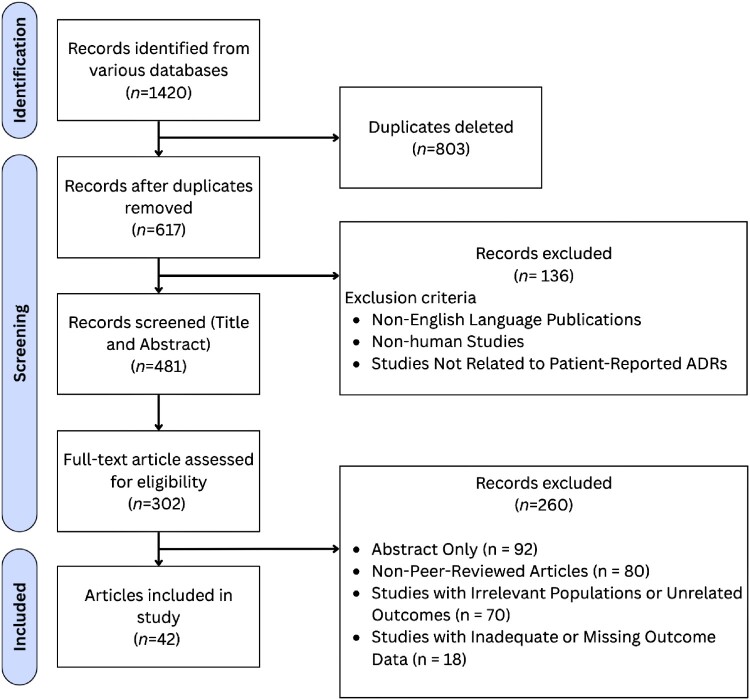
PRISMA flow diagram illustrating the study selection process.

Figure 2 illustrates the study selection process. Originally, 1420 records were identified from various databases. After eliminating 803 duplicates, 617 records remained for screening. These records were further reviewed by title and abstract, resulting in the exclusion of 136 studies based on the initial screening criteria, leaving a total of 481 studies for full-text review. Full-text articles were assessed for eligibility, with 302 articles being reviewed in detail. After applying the exclusion criteria, 260 studies were excluded for reasons such as being abstract-only, non-peer-reviewed, or lacking relevant outcome data. Finally, 42 articles were included in the study.

## Characteristics of included studies

A total of 42 studies were identified and included in this scoping review, and their characteristics are summarised in Table 2. These studies were conducted across various countries, including Canada (Rania Al Dweik, Kohen, et al., 2020; R. Al Dweik, Yaya, et al., 2020), India (Basheera et al., 2022; Kushwaha et al., 2024; Pahuja et al., 2014; Patel et al., 2019; Tejus et al., 2020; Thadani et al., 2019), Nigeria (Adisa et al., 2019), France (Adopo et al., 2022), the UK (Aldeyab et al., 2016; Arnott et al., 2013; Bhoombla et al., 2020), Saudi Arabia (Almubark et al., 2020; Kassem et al., 2021), Ghana (Appiah et al., 2019; Jacobs et al., 2018; Sabblah et al., 2017), Bangladesh (Ata et al., 2021), Turkey (Aydınkarahaliloğlu et al., 2018), Australia (Dedefo et al., 2024; Robertson & Newby, 2013), the Netherlands (de Vries et al., 2021; van Hunsel et al., 2017), Jordan (El-Dahiyat et al., 2023), Bulgaria (Getova et al., 2020), Nepal (Jha et al., 2017), Portugal (Joaquim et al., 2023; Matos et al., 2015), Brazil (Julian et al., 2018), Iraq (Kadhim, 2015), Korea (Kim et al., 2020), South Africa (Matlala et al., 2023), Kenya (Muriithi et al., 2021; Nyagah, 2021), Japan (Noda et al., 2020), Lebanon (Ramia et al., 2021), Pakistan (Rehman et al., 2022), Tanzania (Sirili et al., 2023), Thailand (Srisuriyachanchai et al., 2023) and China (Wang et al., 2023). In terms of study design, the majority of studies employed quantitative descriptive approaches (Adisa et al., 2019; Adopo et al., 2022; Rania Al Dweik, Kohen, et al., 2020; Aldeyab et al., 2016; Almubark et al., 2020; Ata et al., 2021; Aydınkarahaliloğlu et al., 2018; Basheera et al., 2022; Bhoombla et al., 2020; de Vries et al., 2021; Dedefo et al., 2024; Getova et al., 2020; Jha et al., 2017; Joaquim et al., 2023; Julian et al., 2018; Kadhim, 2015; Kim et al., 2020; Kushwaha et al., 2024; Matlala et al., 2023; Matos et al., 2015; Muriithi et al., 2021; Noda et al., 2020; Nyagah, 2021; Pahuja et al., 2014; Patel et al., 2019; Ramia et al., 2021; Rehman et al., 2022; Robertson & Newby, 2013; Sirili et al., 2023; Srisuriyachanchai et al., 2023; Staniszewska et al., 2016; Tejus et al., 2020; Thadani et al., 2019; van Hunsel et al., 2017), while others used qualitative methodologies (R. Al Dweik, Yaya, et al., 2020; Appiah et al., 2019; Arnott et al., 2013; Kassem et al., 2021; Sabblah et al., 2017; Wang et al., 2023) or mixed-methods designs (Jacobs et al., 2018; Nyagah, 2021). The sample sizes ranged from 15 participants (R. Al Dweik, Kohen, et al., 2020; Kassem et al., 2021) to over 500,000 ADR reports from national databases (de Vries et al., 2021; El-Dahiyat et al., 2023; van Hunsel et al., 2017). The most common data collection methods included self-administered questionnaires (El-Dahiyat et al., 2023; Jha et al., 2017; Joaquim et al., 2023; Kadhim, 2015; Kushwaha et al., 2024; Muriithi et al., 2021; Ramia et al., 2021; Tejus et al., 2020; Thadani et al., 2019), semi-structured interviews (R. Al Dweik, Yaya, et al., 2020; Appiah et al., 2019; Arnott et al., 2013; Jacobs et al., 2018; Wang et al., 2023), and electronic surveys (Dedefo et al., 2024; El-Dahiyat et al., 2023). Several studies conducted secondary data analyses of national pharmacovigilance systems such as VigiBase or the Yellow Card Scheme (Adopo et al., 2022; R. Al Dweik, Kohen, et al., 2020; Aydınkarahaliloğlu et al., 2018; de Vries et al., 2021; Matlala et al., 2023; Noda et al., 2020). All studies focused on patients, exploring their roles in direct ADR reporting, their experiences with national pharmacovigilance systems, and perceptions on improving pharmacovigilance strategies. Quality appraisal using the MMAT (2018) indicated that the included studies generally met the core methodological criteria relevant to their design, supporting the robustness of the evidence base.

**Table 2.** Characteristics of included studies.

Author (Year), country	Aim	Sample Size	Study design, data collection, instrument, setting	Results	Conclusions	Barriers	Strategies	Level of awareness and involvement
India (Kushwaha et al., 2024)	To evaluate patients’ awareness and attitudes regarding ADRs and their reporting in a tertiary hospital in Kanpur.	Participants (n = 1389) Male (n = 834) Female (n = 555)	A cross-sectional study using questionnaires was conducted during National Pharmacovigilance Week.	Despite 77.5% of patients indicating they would contact their physician for ADRs, 82.9% were unaware of self-reporting options.	In summary, there is a willingness to report, but low awareness of self-reporting mechanisms remains a major challenge.	Lack of awareness of self-reporting; reliance on physicians.	Public education on self-reporting options.	Low
Nigeria (Adisa et al., 2019)	To assess the knowledge, understanding, attitudes, and practices related to ADR reporting among healthcare professionals and patients in primary healthcare facilities in Ibadan, southwestern Nigeria.	Participants (n = 360)	A cross-sectional survey was conducted using a semi-structured questionnaire that included both open – and closed-ended questions.	Approximately 4.7% of patients had heard of the ADR reporting system, while 95.3% were unaware of its existence.	To conclude, the lack of awareness and understanding requires targeted educational initiatives in primary healthcare settings.	Lack of awareness and inadequate knowledge.	Public education campaigns, training healthcare workers to proactively inform patients.	Low
France (Adopo et al., 2022)	To examine the progression and investigate the factors influencing patient reporting activity in France, with the aim of evaluating patient involvement in pharmacovigilance.	Participants (n = 67,526), Male (n = 13,855), Female (n = 53,671)	A retrospective comparative analysis utilised records from the French National Pharmacovigilance Database (BNPV) and incorporated additional data obtained from the National Institute of Statistics and Economic Studies (INSEE) as well as the Directorate for Research, Studies, Evaluation, and Statistics (DREES).	Patient reporting significantly increased over time in France, particularly among young females, aided by digital tools.	In conclusion, digital innovations have played a vital role in boosting patient participation in pharmacovigilance.	Limited access to online tools for older populations; delay in reporting.	Promote digital tools for ADR reporting and provide assistance for older populations in accessing them.	High
UK (Aldeyab et al., 2016)	To examine patterns in ADR reporting via the Yellow Card (YC) Scheme following the introduction of a patient-oriented YC awareness campaign in community pharmacies across Scotland.	Participants (n = 3610) Patient (n = 275-300)	A cross-sectional study analysing ADR reporting patterns before, during, and after a YC promotional	No statistically significant variation in patient-reported ADR counts was detected following the promotional campaign.	To conclude, campaigns must be tailored to effectively engage the public and sustain interest in pharmacovigilance.	Ineffectiveness of campaign approach.	Increase public engagement with ADR reporting systems; tailor campaigns to emphasise the long-term importance of pharmacovigilance.	Low
Saudi Arabia (Almubark et al., 2020)	To explore the extent of awareness regarding the ADR reporting system.	Participants (n = 5228) Male (n = 2623) Female (n = 2605)	A cross-sectional survey in community pharmacies across Saudi Arabia.	Only 14.3% of participants had ever submitted an ADR report, despite 30.3% being aware of the reporting system, highlighting that awareness alone may not result in active participation.	In conclusion, public awareness should be converted into action by minimising barriers and enhancing the usability of reporting systems.	Lack of awareness of reporting methods, uncertainty of what to report, reliance on HCPs, fear of legal/social issues, and absence of feedback after reporting.	Conduct public campaigns, integrate reporting into mobile apps/pharmacy systems, and train pharmacists to engage patients in discussions on drug safety.	Moderate
Canada (R. Al Dweik, Yaya, et al., 2020)	To investigate patients’ experiences with ADR reporting and their views on the user-friendliness of the Canadian Vigilance reporting forms provided through MedEffect.	Participants (n = 15) Male (n = 2) Female (n = 13)	An interpretive descriptive qualitative study using structured interviews and inductive content analysis to explore patient experiences with ADRs in Canada.	Only 13.3% had used MedEffect to report ADRs; many faced confusion about symptoms and lacked feedback after reporting.	In summary, improving form usability and ensuring follow-up feedback are essential to encourage ongoing patient participation.	Confusion about symptoms linked to ADRs and lack of feedback after reporting.	Increase public awareness; improve ADR reporting forms’ usability and consistency, and ensure follow-up feedback for reporters.	Low
Canada (R. Al Dweik, Yaya, et al., 2020)	To compare consumer – and physician-reported adverse drug reactions (ADRs) based on seriousness, system organ class (SOC), and anatomical therapeutic chemical (ATC) classification.	Participants (n = 198,781) Consumers (n = 57,045)	A retrospective observational study was conducted to analyse ADR reports submitted to the Canadian Vigilance ADR Reporting Database from 2000 to 2014.	Consumers reported significantly more ADRs than physicians, showing strong patient involvement.	In summary, consumer engagement in pharmacovigilance is active and should be supported through continuous awareness efforts.	Consumer reports may be underreported or underestimated in comparison to healthcare professional reports.	Promote continued consumer engagement in ADR reporting through education and awareness campaigns.	High
Ghana (Appiah et al., 2019)	To qualitatively assess the feasibility of using mobile phone caller tunes – the audio or message heard by callers before a call is answered – to encourage patient reporting of ADRs.	Participants (n = 95) Male (n = 70) Female (n = 25)	A qualitative study guided by the Technology Acceptance Model (TAM) as the theoretical framework.	Respondents were open to using mobile phone caller tunes as a reminder for ADR reporting despite existing knowledge gaps.	To conclude, mobile-based tools may be a novel and effective strategy to promote awareness and engagement in ADR reporting.	Lack of knowledge about ADR reporting methods.	Develop mobile phone apps and tools like caller tunes to inform and encourage ADR reporting.	Moderate
UK (Arnott et al., 2013)	To investigate parents’ perspectives and experiences regarding the direct reporting of suspected ADRs in children.	Participants (n = 44) Parents with reporting experience (n = 17) Without (n = 27)	A qualitative study utilised semi-structured interviews with parents, and all interviews were audio-recorded.	Parents, regardless of whether they had reporting experience, were largely unaware of the Yellow Card Scheme, indicating a lack of familiarity with formal ADR processes.	To summarise, parental awareness of formal reporting systems is low, and many assume that healthcare professionals will report on their behalf.	Limited awareness of the Yellow Card Scheme, difficulty identifying ADRs, assumption HCPs would report, complexity of the process, and lack of post-report feedback.	Streamline the reporting process, provide feedback to reporters, educate parents about their rights and responsibilities in ADR reporting.	Low
Bangladesh (Ata et al., 2021)	To evaluate consumers’ awareness of ADR reporting in a tertiary medical college hospital.	Participants (n = 300) Male (n = 123) Female (n = 177)	A descriptive cross-sectional study conducted in a tertiary medical college hospital using self-administered questionnaires administered to a convenience sample of consumers.	Over half (51.11%) of consumers did not report ADRs to anyone; among those who did, most reported to doctors, nurses, or drug sellers.	In summary, many patients remain unaware of the official reporting authority, highlighting the need for awareness on where and how to report ADRs.	Lack of knowledge on where to report ADRs and low engagement with official reporting systems.	Increase awareness of ADR reporting mechanisms and simplify the reporting process for patients.	Low
Turkey (Aydınkarahaliloğlu et al., 2018)	To compare ADR reports from consumers and healthcare professionals.	Participants (n = 6009) Consumers (n = 3141) HCPs (n = 2868)	Retrospective observational study using secondary data submitted to VigiBase between 2014 and 2016.	While HCPs reported more serious ADRs, consumer reports added insights into less severe reactions and contributed valuable perspectives.	In summary, increasing consumer education can enhance their role in reporting a broader range of ADRs.	Lack of consumer participation in ADR reporting; consumers may not recognise less severe ADRs as reportable.	Increase consumer awareness and engagement in ADR reporting; improve consumer education on recognising ADRs.	Low
India (Basheera et al., 2022)	To assess the knowledge and awareness of ADRs and the pharmacovigilance system among outpatients attending various departments of a super-specialty hospital.	Participants (n = 50) Male (n = 27) Female (n = 23)	A cross-sectional observational study was conducted at a multi-specialty hospital, with randomly selected outpatients surveyed using a bilingual questionnaire.	Only 4% had heard of the PvPI ADR app through social media, while 96% were unaware of its existence	To conclude, hospital-based educational campaigns are necessary to promote the use of mobile ADR reporting tools.	Lack of awareness of ADR reporting tools; difficulty in reporting due to hospital rush.	Develop targeted educational campaigns in hospitals and promote the ADR PvPI app for self-reporting.	Low
UK (Bhoombla et al., 2020)	To examine how children and young people (CYP) contribute to the Medicines and Healthcare Products Regulatory Agency’s (MHRA) Yellow Card Scheme (YCS).	Participants (n = 41,630) Children <19 years (n = 948)	A retrospective review of suspected ADR reports submitted to the MHRA Yellow Card Scheme (YCS) from 2008 to 2018.	Only 2.3% of total Yellow Card Scheme reports came from children and young people.	In summary, although children can contribute to signal detection, their involvement remains limited and needs improvement.	Limited involvement from younger age groups.	Improve outreach to CYP and their families; educate healthcare providers to engage this group in reporting ADRs.	Moderate
Australia (Dedefo et al., 2024)	To explore Australian consumers’ current knowledge and experiences regarding ADR reporting, along with their reasons for reporting or not reporting ADRs, with particular emphasis on the use of digital tools for reporting.	Participants (n = 544) Male (n = 113) Female (n = 370)	A cross-sectional online survey was administered to adults in Australia who had taken medication.	Only 36% of participants had reported ADRs, and 58% of those were unaware that digital tools could be used for reporting.	In conclusion, digital reporting tools remain underutilised, and awareness campaigns should focus on increasing knowledge about these platforms.	Lack of awareness of digital tools and low engagement with reporting channels.	It is important to enhance education on the use of digital tools for ADR reporting and promote greater integration of reporting systems within healthcare applications.	Moderate
Croatia, Netherlands, UK (de Vries et al., 2021)	To evaluate the extent to which the motives for reporting ADRs differ between healthcare professionals (HCPs) and patients across countries.	Participants (n = 459) Croatia (*n* = 136) Male (n = 39) Female (n = 97) Netherlands (*n* = 187) Male (n = 91) Female (n = 96) UK (*n* = 98) Male (n = 29) Female (n = 69)	A cross-sectional web-based survey conducted under the IMI Web-RADR project and the SCOPE Joint Action.	UK patients were most aware of ADR reporting systems, followed by the Netherlands and Croatia, yet many are unaware of how to report or what happens afterward.	In summary, there is a general need to improve patient understanding of the reporting process across multiple countries.	There is a lack of awareness of steps to report ADRs, along with confusion about the reporting process.	Improve digital engagement tools and awareness campaigns for ADR reporting, particularly for patients in Croatia and the Netherlands.	Moderate
Jordan (El-Dahiyat et al., 2023)	To assess the knowledge, attitudes, and practices of the general public in Jordan regarding ADR reporting and PV.	Participants (n = 441) Male (n = 143) Female (n = 298)	A cross-sectional study was conducted using a four-section electronic survey administered to a convenience sample of Jordanian participants.	Although 70.3% of patients believed ADRs should be reported, only 8.1% actually reported them to the national pharmacovigilance centre.	To conclude, public education campaigns and improved visibility of the reporting system are needed to increase reporting rates.	Low public awareness of the existence or function of the JNCP.	Promote the ADR reporting system via education, provide direct communication with healthcare providers, and engage the public through media campaigns.	High
Bulgaria (Getova et al., 2020)	To assess patients’ knowledge of pharmacovigilance, with particular emphasis on additional monitoring.	Participants (n = 316) Male (n = 117) Female (n = 199)	Cross-sectional study using closed-ended validated questionnaires.	Around 31.3% of patients were aware of direct ADR reporting to national authorities, and only 21.5% understood the black triangle symbol.	In conclusion, more efforts are needed to educate the public on pharmacovigilance markers and reporting options.	No data.	No data.	Low
Ghana (Jacobs et al., 2018)	To assess Ghanaian patients’ knowledge of ADRs and the reporting process, as well as to investigate how they recognise an ADR and the steps they take after experiencing it.	Participants (n = 560) Male (n = 278) Female (n = 293)	A mixed-methods design, consisting of a quantitative survey and qualitative in-depth interviews.	Although 38% had experienced an ADR, only 3% were aware of the Ghana FDA’s reporting system, with the majority reporting solely to doctors.	In conclusion, many Ghanaian patients seem to be unaware of, unable to access, or unwilling to use formal national channels for ADR reporting, such as the Ghana FDA’s Patient Reporting System (PRS).	Lack of awareness about the national pharmacovigilance system (PRS).	Increase patient awareness of the Ghana FDA’s functions and encourage utilisation of the PRS for ADR submissions.	Low
Nepal (Jha et al., 2017)	To examine consumers’ knowledge, attitudes, and practices related to pharmacovigilance and consumer pharmacovigilance in Nepal’s Lalitpur district.	Participants (n = 157) Male (n = 67) Female (n = 90)	A cross-sectional study using questionnaires was conducted among outpatients at KIST Medical College and Teaching Hospital in Lalitpur, Nepal.	As low as 2.54% of patients were aware that Nepal’s Department of Drug Administration serves as the national pharmacovigilance centre.	In summary, patient education on pharmacovigilance and the role of national authorities must be prioritised.	Lack of awareness of DDA and limited knowledge about pharmacovigilance programmes.	Increase education on pharmacovigilance, highlight the role of DDA in ADR reporting.	Low
Portugal (Joaquim et al., 2023)	To assess patients’ awareness of ADR risks and their knowledge of the Portuguese PV system.	Participants (n = 91) Male (n = 30) Female (n = 61)	A cross-sectional survey employing a 27-item questionnaire was carried out at a health centre in Coimbra, Portugal.	Although patients had satisfactory knowledge of pharmacovigilance, 82.4% were unaware of the national system and lacked the practical knowledge to report.	In summary, improving health literacy and awareness of reporting mechanisms is crucial to boost patient engagement.	Patients demonstrated limited awareness of the procedures for reporting ADRs.	Improve health literacy and education.	Low
Brazil (Julian et al., 2018)	To assess knowledge and perceptions regarding pharmacovigilance in Brazil.	Participants (n = 263)	A descriptive, cross-sectional, web-based survey was conducted between April 2012 and October 2014.	Only 7.2% knew adverse events could be reported to pharmaceutical companies; most reported to physicians instead.	To conclude, gaps in pharmacovigilance knowledge, especially in oncology patients, require targeted educational outreach.	Lack of awareness about reporting mechanisms; limited engagement with the ADR reporting system.	Targeted educational initiatives for oncology patients to increase knowledge about pharmacovigilance.	Low
Iraq (Kadhim, 2015)	To assess patients’ knowledge and attitudes regarding ADR reporting in Baghdad.	Participants (n = 300) Male (n = 165) Female (n = 135)	A cross-sectional observational study conducted in outpatient general hospitals.	Although 73.3% of patients were aware of ADRs and 37% had experienced them, none knew of an ADR reporting centre.	In summary, structured educational interventions are necessary to bridge the awareness gap and encourage reporting behaviours.	There was a lack of awareness regarding ADR reporting centres, coupled with the absence of structured educational programmes on how to report ADRs.	Establish an ADR reporting infrastructure and promote education programmes within hospitals to inform and encourage patients to report ADRs..	Low
Saudi Arabia (Kassem et al., 2021)	To investigate community perspectives and the need for patient-friendly smartphone applications (SPAs) to facilitate greater participation in ADR reporting.	Participants (n = 15) Male (n = 6) Female (n = 9)	A qualitative study employing purposeful sampling to recruit participants, with data collected through semi-structured interviews.	All participants were unaware of the Saudi FDA-ADR system and had no prior education on ADR reporting.	In summary, education and user-friendly digital platforms are urgently needed to engage the public in ADR reporting.	Patients in Saudi Arabia have very low awareness and knowledge of ADR reporting systems, with no prior exposure to the Saudi FDA-ADR platform.	Develop patient-friendly smartphone apps; education programmes.	Low
Korea (Kim et al., 2020)	To evaluate consumers’ knowledge, attitudes, and intentions regarding ADR reporting, and to investigate factors influencing their reporting intentions in South Korea.	Participants (n = 1000) Male (n = 509) Female (n = 491)	A cross-sectional survey employing self-administered questionnaires, distributed to a nationwide convenience sample of consumers with regional stratification.	Although 59.2% of participants expressed willingness to report ADRs, only 3.4% had actually reported, and fewer than 15% were aware of the self-reporting system.	Therefore, the intent to report exists, but knowledge and experience of self-reporting systems remain inadequate.	Lack of awareness of self-reporting systems; lack of direct reporting experience.	Raise awareness of self-reporting systems; increase educational programmes on ADR reporting.	Low
South Africa (Matlala et al., 2023)	To characterise the demographic and clinical profiles of spontaneous ADR reports received by SAHPRA in 2017.	Participants (n = 8438) Male (n = 2790) Female (n = 5227)	A retrospective cross-sectional study utilising ADR data from VigiBase, the WHO global ICSR database, analysing demographics, report completeness, and clinical details.	Physicians submitted 39.66% of ADR reports, while patients submitted 29.39%, but many were incomplete.	In summary, patients play a valuable role in ADR reporting, but training is needed to improve report quality.	Missing clinical data and a need for reporter training.	Provide training for all healthcare providers and consumers; improve reporting systems to ensure completeness of ADR data.	Moderate
Portugal (Matos et al., 2015)	To examine patients’ knowledge and attitudes toward spontaneous ADR reporting, as well as the reasons and perceptions that may contribute to underreporting.	Participants (n = 948)	A descriptive-correlational study was carried out among general adult patients at a community pharmacy in Coimbra, Portugal.	A large number of patients were unaware of direct ADR reporting systems and preferred reporting through healthcare professionals.	In summary, there is a preference for indirect reporting, which reflects the need to empower patients with direct reporting knowledge.	Lack of awareness about direct ADR reporting systems; preference for reporting through healthcare professionals.	Increase awareness of direct ADR reporting; educate patients on their rights and responsibilities.	Low
Kenya (Muriithi et al., 2021)	To determine factors influencing ADR reporting among patients.	Participants (n = 360) Male (n = 112) Female (n = 248)	Cross-sectional study within four selected health facilities in Kirinyaga County, using pretested interviewer-administered questionnaires.	While 74.4% felt it was their responsibility to report ADRs, 73.3% were unaware of tools like the patient alert card.	To conclude, increasing awareness of ADR tools is essential to enable patients to take responsibility for pharmacovigilance.	Lack of awareness about ADR reporting tools; reluctance to report ADRs.	Increase awareness of ADR tools; provide education on patient reporting responsibilities.	Moderate
Japan (Noda et al., 2020)	To assess ADR reports involving pediatric patients in the JADER database.	Participants (n = 504,407) Children aged <10 and 10–19 years (n = 21,359)	A retrospective study analysing spontaneous ADR reports for pediatric patients included in the JADER database from 2004 to 2017.	More than 50% of ADR reports were for children under 10, with fatal outcomes observed in 4.7% (<10 years) and 3.9% (10–19 years).	To conclude, continuous and careful monitoring of ADRs in pediatric populations is necessary to ensure drug safety.	No data.	Improve monitoring systems for pediatric ADRs; conduct targeted studies to understand specific ADR risks in children.	High
Kenya (Nyagah, 2021)	To investigate factors influencing ADR reporting among patients and healthcare providers in selected hospitals in Kirinyaga County.	Participants (n = 166)	A mixed-method study; interviewer-administered and self-administered questionnaires; stratified and purposive sampling. SPSS v27 was used for analysis.	Among patients who experienced ADRs, 87.3% reported them to healthcare providers, yet formal engagement with pharmacovigilance tools was limited.	To conclude, while awareness exists, tools and knowledge for formal ADR reporting must be improved.	Limited awareness of pharmacovigilance tools and reluctance to report ADRs.	Promote education about ADR reporting tools; provide better communication about the importance of reporting.	Moderate
India (Pahuja et al., 2014)	To assess the level of consumer and patient awareness regarding the adverse drug reaction reporting system in India.	Participants (n = 770)	A cross-sectional study was carried out among patients admitted to the All India Institute of Medical Sciences, New Delhi.	Only 4% of patients were aware of the PvPI, with most preferring to report ADRs through hospital drop boxes rather than by phone.	In summary, patient knowledge about the ADR reporting system is poor, necessitating better communication channels.	Low awareness and poor knowledge.	Conduct pilot studies to evaluate the effectiveness of various ADR reporting methods, including internet, postal, and telephonic approaches.	Low
India (Patel et al., 2019)	To assess the knowledge and attitude of consumers regarding ADR reporting and observe the practice of ADR reporting.	Participants (n = 820) Male (n = 425) Female (n = 395)	A cross-sectional hospital-based survey.	Only 1.2% were aware of the PvPI, yet 96.1% believed that direct consumer reporting could help improve pharmacovigilance.	To conclude, the gap between belief and awareness must be addressed through widespread education and outreach.	There was a lack of knowledge and awareness regarding the ADR reporting system and the Pharmacovigilance Programme of India (PvPI).	Implement public awareness campaigns to educate patients about the ADR reporting system and the Pharmacovigilance Programme of India (PvPI).	Low
Lebanon (Ramia et al., 2021)	To assess the prevalence and determinants of self-reported ADEs among Lebanese outpatients.	Participants (n = 3109) Male (n = 1550) Female (n = 1559)	A cross-sectional, questionnaire-based study conducted among Lebanese outpatients visiting a community pharmacy between March and May 2016.	A total of 70.5% of patients reported adverse drug events (ADEs) to their physicians. However, 17% were unable to contact a physician, 16% lacked knowledge about ADR reporting, and 14.8% felt that physicians were unapproachable.	To conclude, patient education on ADR reporting is essential to enhance prevention, increase awareness, and support regulatory decisions.	Inability to reach physician, lack of education on ADR reporting and physician unapproachability.	Educate patients about the importance of reporting, improve communication with HCPs, and establish active national pharmacovigilance centres.	Moderate
Pakistan (Rehman et al., 2022)	To evaluate patients’ perceptions of ADRs and examine the effects of a health promotion intervention.	Participants (n = 423) Male (n = 193) Female (n = 230)	A pre – and post-intervention cross-sectional survey was conducted in public tertiary care hospitals in Islamabad, Pakistan, incorporating a health promotion intervention that included educational brochures and pharmacist-led counselling sessions.	Post-intervention, intention to report increased from 57.2% to 99.3%, and awareness of pharmacovigilance centres rose from 56.7% to 97.9%.	To conclude, pharmacist-led counselling and brochures are highly effective in improving patient knowledge and engagement in pharmacovigilance.	Low baseline awareness of pharmacovigilance and ADR reporting mechanisms; limited communication between patients and healthcare providers.	Distribute educational brochures and provide pharmacist-led counselling to improve ADR reporting awareness.	High
Australia (Robertson & Newby, 2013)	To assess the level of public awareness regarding consumer ADR reporting systems in Australia.	Participants (n = 4981)	A cross-sectional study was carried out using computer-assisted telephone interviews (CATI) alongside a national sample completing an online Pureprofile survey.	Only 10.4% of respondents were aware of consumer ADR reporting schemes in Australia, while 87.5% had no knowledge of them..	In conclusion, public awareness of ADR reporting systems is low, highlighting the need for nationwide educational and awareness initiatives..	Lack of awareness and low engagement with pharmacovigilance systems.	Public awareness campaigns are needed to inform the public about the availability of ADR reporting systems.	Low
Ghana (Sabblah et al., 2017)	To investigate patients’ knowledge, attitudes, behaviours, and perceptions regarding spontaneous ADR reporting in Ghana.	Participants (n = 434) Male (n = 210) Female (n = 224)	A cross-sectional survey design using structured questionnaires in selected healthcare facilities in Ghana.	Although 81.6% of participants were aware of the national centre, only 49.5% knew they could report directly, and many expressed concerns about imposing on healthcare professionals.	In summary, while awareness is moderate, simplifying the reporting process and encouraging open communication can increase reporting rates.	Lack of awareness of how to report ADRs; fear of burdening healthcare providers.	Establish clearer reporting processes; encourage healthcare providers to proactively inform patients about ADR reporting.	Moderate
Tanzania (Sirili et al., 2023)	To evaluate patients’ awareness, behaviours, and factors influencing their actions regarding ADR reporting at a referral hospital in the Southern Highlands of Tanzania.	Participants (n = 792) Male (n = 397) Female (n = 395)	A hospital-based cross-sectional study was carried out from January to August 2022 at Mbeya Zonal Referral Hospital (MZRH) in Mbeya, Tanzania.	Although 70.3% of participants indicated they would report ADRs to healthcare providers, 69.1% were unaware of the significance of ADR reporting.	In conclusion, enhancing patients’ understanding of the importance of ADR reporting is crucial.	Limited knowledge of ADRs and their significance.	Educate patients on ADRs and the advantages of reporting, while promoting effective communication with healthcare providers.	Low
Thailand (Srisuriyachanchai et al., 2023)	To compare ADR severity levels as rated by patients and pharmacists, and to examine the methods used by both patients and healthcare professionals for ADR management and prevention.	Participants (n = 419), Male (n = 141), Female (n = 278)	A cross-sectional study using a self-administered questionnaire and medical record review.	Most patients responded to ADRs by consulting a physician (67.5%), stopping the drug (45.4%), or continuing at the same dose (9.9%), but their formal reporting behaviours remained unclear.	In summary, while patients do take action upon experiencing ADRs, more focus is needed on encouraging formal reporting practices.	No data.	No data.	Moderate
Poland (Staniszewska et al., 2016)	To assess patient knowledge on the reporting of ADR.	Participants (n = 200) Male (n = 24) Female (n = 176)	A prospective cross-sectional survey study.	Although 90% believed doctors could report ADRs and 75% said patients could too, there was confusion and low awareness about how to report.	To conclude, there is a need to clarify patient rights and responsibilities in ADR reporting through education.	There was a lack of awareness about ADR reporting systems and uncertainty regarding patients’ reporting rights.	Increase education about ADR reporting rights and systems.	Low
India (Tejus et al., 2020)	To evaluate patients’ knowledge, attitudes, and practices regarding the PvPI.	Participants (n = 523) Male (n = 305) Female (n = 218)	Cross-sectional observational study using an interviewer-administered questionnaire.	Only 10% of patients were aware of the PvPI and its helpline number.	To conclude, awareness about national pharmacovigilance systems is extremely low and must be strengthened through education.	No data.	No data.	Low
India (Thadani et al., 2019)	To evaluate patients’ knowledge and awareness of ADRs and the associated reporting processes.	Participants (n = 172) Male (n = 63) Female (n = 109)	An observational cross-sectional study using a questionnaire was conducted in a tertiary care hospital.	Of the individuals who experienced ADRs, 67.5% reported them to a healthcare professional; however, awareness of reporting systems was still limited.	In summary, better education is needed to bridge the gap between willingness and effective use of reporting systems.	Low awareness of reporting systems and difficulty in identifying ADRs.	Enhance public education and awareness; simplify the ADR reporting process.	Low
Netherlands (van Hunsel et al., 2017)	To evaluate the current role of patient reports in disseminated signals to the Dutch Medicines Evaluation Board and identify whether certain types of signals show a unique contribution from patient reports compared to those from HPCs and marketing authorisation holders.	Participants (n = 1692) Patients (n = 445)	A quantitative, cross-sectional, retrospective analysis of patient reports submitted to the Netherlands Pharmacovigilance Centre (Lareb) between 2010 and 2015.	Patient reports made distinct contributions, particularly in cases involving drug substitution, accounting for 6.5% of such reports.	To conclude, patient reporting is valuable and should be encouraged, especially where unique insights can support signal detection.	Low patient involvement in the reporting system.	Educate patients about their role in pharmacovigilance and the importance of their reports.	Moderate
China (Wang et al., 2023)	To explore how elderly patients with chronic conditions identify and cope with ADEs.	Elderly Patients (n = 15), Male (n = 8), Female (n = 7)	Qualitative semi-structured interviews using purposive sampling, at a medical centre in northern China.	Elderly patients demonstrated low awareness and poor participation in ADR monitoring, primarily due to limited access to information and weak communication with healthcare providers.	To conclude, empowering elderly patients through better access to information and improved communication is crucial for enhancing their reporting behaviour.	Poor awareness, limited information access, communication difficulties with HCPs, low health literacy, and lack of confidence to report	Tailored educational programmes for the elderly, enhanced doctor-patient communication, and development of accessible tools for seniors.	Low

Abbreviations: ADR, adverse drug reaction; ADE, adverse drug event; ATC, Anatomical Therapeutic Chemical classification; BNPV, Base Nationale de Pharmacovigilance; CATI, computer-assisted telephone interview; CYP, children and young people; DDA, Department of Drug Administration; DREES, Directorate for Research, Studies, Evaluation, and Statistics; FDA, Food and Drug Administration; HCP, healthcare professional; ICSR, individual case safety report; IMI, Innovative Medicines Initiative; INSEE, Institut National de la Statistique et des Études Économiques; JADER, Japanese Adverse Drug Event Report database; JNCP, Jordan National Pharmacovigilance Center; KIST, KIST Medical College and Teaching Hospital; MAH, marketing authorisation holder; MHRA, Medicines and Healthcare products Regulatory Agency; MZRH, Mbeya Zonal Referral Hospital; PRS, Patient Reporting System; PV, pharmacovigilance; RADR, Recognising Adverse Drug Reactions; SAHPRA, South African Health Products Regulatory Authority; SCOPE, Strengthening Collaboration for Operating Pharmacovigilance in Europe; SOC, System Organ Class; SPSS, Statistical Package for the Social Sciences; TAM, Technology Acceptance Model; WHO, World Health Organization; YC, Yellow Card; YCS, Yellow Card Scheme.

## Level of patient awareness on ADRs

Across the reviewed literature, patient awareness of ADRs exhibited considerable variability, although it was generally assessed as low to moderate. Numerous studies revealed that a substantial proportion of patients lacked fundamental knowledge regarding the definition of ADRs, the severity levels, and the significance of reporting them.

For instance, findings from Jordan (El-Dahiyat et al., 2023), Iraq (Kadhim, 2015), and Ghana (Sabblah et al., 2017) consistently showed that most participants had never heard of formal ADR reporting systems. In contrast, some studies conducted in the UK [17], India [39], and the Netherlands [49] reported comparatively higher levels of awareness, often linked to education campaigns or national pharmacovigilance initiatives. Interestingly, several studies noted that patients could identify and describe personal experiences of ADRs, even if they did not categorise them using formal terminology. For example, participants in qualitative studies from Saudi Arabia (Kassem et al., 2021) and China (Wang et al., 2023) provided vivid accounts of adverse symptoms they had experienced, yet did not associate these with a need to report. This disconnection between experiential knowledge and formal reporting practices highlights a key gap in pharmacovigilance education and communication.

## Patient involvement in ADR reporting

Despite growing efforts to promote patient participation in ADR reporting, actual involvement remains relatively low across most countries. Quantitative studies from Nigeria (Adisa et al., 2019), Turkey (Aydınkarahaliloğlu et al., 2018), and Nepal (Jha et al., 2017) reported that less than 20% of respondents had never reported an ADR. In contrast, higher levels of participation were observed in studies from the UK (Arnott et al., 2013), Australia (Robertson & Newby, 2013), and the Netherlands (van Hunsel et al., 2017), particularly in contexts where patient-centred pharmacovigilance tools such as online reporting portals and mobile applications were available. Several studies demonstrated a positive correlation between awareness and reporting behaviour. For instance, research conducted in Portugal (Joaquim et al., 2023) and South Africa (Matlala et al., 2023) indicated that patients who had received counselling from healthcare professionals or encountered health promotion materials were significantly more likely to submit reports. Additionally, qualitative data from Bangladesh (Ata et al., 2021) and Kenya (Muriithi et al., 2021) revealed that patients who experienced severe or frightening symptoms felt more compelled to report, even in the absence of structured systems.

## Barriers to patient reporting of ADRs

A wide range of barriers affecting patient reporting behaviour were identified across the studies. One of the most frequently cited obstacles was a lack of knowledge regarding the existence and purpose of ADR reporting systems. For example, in Saudi Arabia, a study found that more than half of the participants were unaware of any formal mechanism for ADR reporting (Almubark et al., 2020). Similarly, in India, a low engagement due to a perceived lack of relevance or urgency (Kushwaha et al., 2024; Thadani et al., 2019). Practical challenges also played a role. Patients in Ghana (Appiah et al., 2019) and Brazil (Julian et al., 2018) described the ADR reporting process as overly complicated, citing difficulties with form completion, lack of clear instructions, and the use of medical jargon. These concerns were echoed in the UK study, where even participants with relatively high health literacy expressed confusion about how and where to report (Bhoombla et al., 2020). Moreover, psychosocial factors also emerged as important, with many patients expressing uncertainty about whether their symptoms qualified as ADRs, and others fearing blame or negative consequences for submitting inaccurate reports. In qualitative research from Canada (Rania Al Dweik, Kohen, et al., 2020), patients reported feelings of uncertainty about whether their symptoms qualified as ADRs, and some expressed fear of being blamed for incorrect medicine use. Additionally, limited feedback following reports discouraged further participation, as seen in studies from France (Adopo et al., 2022) and Japan (Noda et al., 2020). These findings point to the need for more supportive and user-friendly reporting environments that build confidence and trust.

## Motivators and facilitators for ADR reporting

On the other hand, several motivating factors were also reported that encouraged patients to engage in ADR reporting. Across both quantitative and qualitative studies, a consistent finding was the desire to prevent harm to others. Participants in studies from South Africa (Matlala et al., 2023), Australia (Robertson & Newby, 2013), and Thailand (Srisuriyachanchai et al., 2023) expressed a sense of responsibility to inform authorities about their adverse experiences so that others might be protected. Other motivators included positive reinforcement from healthcare providers, accessible and simplified reporting platforms, and assurances that patient voices are valued.

In Portugal (Joaquim et al., 2023), the study found that personalised acknowledgment after a report submission increased satisfaction and the likelihood of future engagement. Similarly, studies from Korea (Kim et al., 2020) and Pakistan (Rehman et al., 2022) noted that patients were more likely to report ADRs when they had access to online platforms that provided confirmation messages or feedback.

## Sources of information on ADRs

Multiple sources of information regarding ADR reporting were identified across the studies, with healthcare professionals being the most frequently cited. Patients in Nigeria (Adisa et al., 2019), Lebanon (Ramia et al., 2021), and Jordan (El-Dahiyat et al., 2023) reported that physicians and pharmacists were their primary sources of information about ADRs. Additionally, studies from the UK (Arnott et al., 2013) and the Netherlands (de Vries et al., 2021) showed an increasing reliance on digital tools such as websites, mobile apps, and social media campaigns. In many cases, patient exposure to pharmacovigilance information was incidental, occurring during outpatient visits or medication counselling sessions. In contrast, deliberate awareness campaigns such as posters, radio announcements, or national PV week events were less commonly encountered, particularly in LMICs. This suggests that patient education on ADR reporting is often informal and inconsistent, potentially contributing to the observed variability in awareness and participation.

## Platforms and channels for patient ADR reporting

Studies demonstrated a wide variation in the platforms and channels available for patients to report ADRs. In high-income countries such as the UK, Sweden, and Australia, national regulatory agencies have developed comprehensive online portals and mobile applications to facilitate direct patient reporting (Arnott et al., 2013; Robertson & Newby, 2013). In these contexts, patients reported relatively higher satisfaction with the ease and convenience of reporting. However, in LMICs, reporting mechanisms were often underdeveloped. Paper-based reporting and in-person submissions remained the most common modes, as seen in Nigeria (Adisa et al., 2019), Nepal (Jha et al., 2017), and Tanzania (Sirili et al., 2023). In such settings, a lack of internet access, technological infrastructure, and trained personnel further limited reporting opportunities. Recent innovations such as SMS-based alerts and automated call systems have shown potential in improving accessibility.

In India, it was reported that the PVPI launched a mobile app to encourage patient involvement, leading to a significant increase in report submissions [20].

## Strategies to improve patient awareness and engagement

Numerous studies proposed targeted interventions to improve patient awareness and participation in ADR reporting. Educational programmes emerged as one of the most widely recommended strategies. In India, (Kushwaha et al., 2024; Pahuja et al., 2014) found that structured pharmacovigilance education in both community and hospital settings significantly improved knowledge levels and intent to report. Similarly, in Ghana, (Sabblah et al., 2017) reported increased reporting rates following public sensitisation activities conducted by the FDA. Digital platforms were widely recognised across several included studies as promising tools to improve patient engagement in ADR reporting. For instance, a study in Pakistan (Rehman et al., 2022) reported that web-based and mobile reporting systems provided more convenient alternatives to conventional paper forms, particularly in regions with growing access to digital technologies. Although most studies did not describe platform design in detail, mobile apps and online portals were generally seen as helpful in reducing barriers and simplifying the reporting process, particularly in high mobile-use settings. In addition, several studies emphasised the importance of healthcare professionals in promoting patient engagement. For example, research conducted in Saudi Arabia (Kassem et al., 2021), Nepal (Jha et al., 2017), and South Africa (Matlala et al., 2023) found that patients were more likely to report ADRs when encouraged or supported by pharmacists or physicians. Hence, integration of ADR reporting prompts during consultations and follow-up visits was also suggested as a means of reinforcing patient involvement in real-time.

## Socioeconomic and demographic factors on ADR awareness and reporting

Socioeconomic and demographic variables were consistently shown to influence ADR reporting behaviour across the studies. Age, education level, income, and urban or rural residence all appeared to play roles in determining awareness and likelihood of participation. A study in Lebanon (Ramia et al., 2021) reported that younger, urban, and university-educated individuals were significantly more likely to be aware of ADR reporting systems (Ramia et al., 2021). A similar trend was observed in Kenya (Muriithi et al., 2021), where respondents with higher levels of formal education reported greater familiarity with pharmacovigilance. Furthermore, gender differences were also noted in some studies. For instance, a study found that women were more likely to report ADRs compared to male gender, possibly due to differences in health-seeking behaviour and communication styles (van Hunsel et al., 2017). However, this finding was not consistent across all settings. In addition, studies conducted in Nigeria (Adisa et al., 2019) and Thailand (Srisuriyachanchai et al., 2023) reported no significant differences in ADR reporting behaviour between male and female patients, suggesting that gender may not be a determining factor in patient engagement in these settings. Access to healthcare resources was another key factor. Studies from Brazil (Julian et al., 2018) and Tanzania (Sirili et al., 2023) suggested that limited interaction with the healthcare system, due to geographic or financial barriers, reduced patient exposure to pharmacovigilance information and therefore diminished reporting. These findings indicate the need for targeted awareness strategies that consider the diverse demographic and socioeconomic realities of different populations.

## Discussion

The main finding of this scoping review is that patient awareness and involvement in ADR reporting remain consistently low despite ongoing global efforts to strengthen pharmacovigilance. Across the reviewed studies, a considerable proportion of patients demonstrated limited understanding of what constitutes an ADR, the seriousness of its impact, and how to report such reactions to national authorities. This issue is particularly prominent in LMICs where structured health education is insufficient and access to pharmacovigilance systems is often constrained. For instance, studies from Iraq, India, Nepal, and Nigeria highlighted widespread unfamiliarity with pharmacovigilance centres, reporting mechanisms, and the role of patients in post-marketing drug safety surveillance (Adisa et al., 2019; Jha et al., 2017; Kadhim, 2015; Kushwaha et al., 2024). Although patients in some regions acknowledged the importance of recognising ADRs, they often assumed that reporting should be handled exclusively by healthcare professionals (Arnott et al., 2013; Matos et al., 2015; Pahuja et al., 2014).

In contrast, populations in countries like France, Australia, and Portugal showed relatively higher awareness, which could be attributed to structured public health campaigns and the integration of digital resources into national pharmacovigilance frameworks (Adopo et al., 2022; Joaquim et al., 2023; Robertson & Newby, 2013). France’s reporting platform (signalement-sante.gouv.fr) and Australia’s *MedSearch* and TGA websites were designed to be accessible and easy to navigate, thereby encouraging more patient engagement (Adopo et al., 2022; Joaquim et al., 2023). Despite increased awareness in some regions, direct patient reporting still remains limited. Many patients prefer to report adverse reactions to physicians or pharmacists, rather than through formal digital or paper-based systems (Getova et al., 2020; Ramia et al., 2021; Tejus et al., 2020). This heavy reliance on healthcare intermediaries often results in underreporting, especially when those professionals are overwhelmed or unaware of the importance of consumer-submitted reports. Nonetheless, the introduction of user-friendly technologies has begun to bridge this gap. Furthermore, Saudi Arabia’s SFDA-ADR mobile application and India’s PvPI Mobile App were introduced to simplify the submission process and enable real-time reporting by both patients and healthcare providers (Rehman et al., 2022; Thadani et al., 2019).

In addition, although there has been a significant advancement in digital tools, their effectiveness remains dependent on awareness and digital literacy. In many countries, especially those with limited infrastructure or high illiteracy rates, patients remain unaware that such platforms exist or lack the skills to use them effectively (Aldeyab et al., 2016; Matlala et al., 2023). Furthermore, the absence of feedback following report submission, as reported by Pahuja et al. (2014), led many patients to feel that their contributions had little or no value (Pahuja et al., 2014). This review identified multifaceted barriers to patient reporting. In addition to lack of knowledge, patients expressed uncertainty about whether their symptoms qualified as ADRs and whether it was appropriate for them to report such observations independently. Others feared blame or legal consequences if the report was deemed inaccurate. In settings where pharmacovigilance systems were unfamiliar or underdeveloped, the technical language used in reporting forms further discouraged participation (Jha et al., 2017; Kushwaha et al., 2024; Matlala et al., 2023).

In particular, drug information leaflets were frequently cited as difficult to read or understand due to their complex structure and terminology. In response, several studies recommended the use of simplified, culturally adapted leaflets written in local languages to enhance comprehension and encourage safer medicine use (Joaquim et al., 2023; van Hunsel et al., 2017). In contrast, several motivators for patient involvement in ADR reporting were also identified. Notably, patients who had personally experienced an ADR and had been previously acknowledged by a healthcare provider or regulatory body were more likely to report subsequent events (Adopo et al., 2022; Sirili et al., 2023). Educational campaigns, pharmacist-led counselling, and awareness activities conducted during national pharmacovigilance weeks significantly improved public engagement in countries such as Pakistan, Saudi Arabia, and Ghana (Getova et al., 2020; Jacobs et al., 2018; Sirili et al., 2023). Moreover, platforms that provided clear guidance and minimal steps to complete reports were preferred. For instance, the Netherlands’ *Lareb* platform and the UK’s *Yellow Card Scheme* website (*yellowcard.mhra.gov.uk*) were both noted in the literature as effective tools that empowered patients to report directly and conveniently (de Vries et al., 2021; Pahuja et al., 2014).

The source of ADR information greatly influenced reporting behaviour. Most patients relied on healthcare providers, particularly pharmacists and physicians, as their primary source of drug safety information (Getova et al., 2020; Kim et al., 2020; Ramia et al., 2021). However, the quality and consistency of this information varied across settings. In some cases, healthcare professionals lacked the time or initiative to adequately educate patients about ADRs. In addition to clinical encounters, mass media campaigns, posters in community pharmacies, and digital health messages delivered via mobile phones were identified as effective communication channels in India (Basheera et al., 2022), Ghana (Jacobs et al., 2018), and Thailand (Srisuriyachanchai et al., 2023). Despite these outreach efforts, drug leaflets remain underutilised, particularly in communities where literacy rates are low. Several studies proposed reformatting these materials using visual aids and plain language to improve accessibility. Furthermore, some regions explored innovative tools such as caller tunes and mobile push notifications to raise awareness, as exemplified by a qualitative study in Ghana, which found that mobile audio alerts could potentially increase engagement with pharmacovigilance efforts (Appiah et al., 2019).

Reporting channels varied considerably across countries. In India and Nepal, hospital drop-box systems and physical reporting forms remained common due to limited internet access (Jha et al., 2017; Tejus et al., 2020). Although internet penetration and digital literacy in India have improved in recent years, some rural and remote areas may still face challenges in accessing online ADR reporting systems(Shukla et al., 2024).

However, in high-income countries, the rise of mobile applications and national web portals has provided patients with accessible alternatives such as Canada’s *MedEffect Canada* reporting form and Portugal’s *INFARMED* platform (R. Al Dweik, Yaya, et al., 2020; Joaquim et al., 2023). Although adoption of these tools is gradually increasing, their use remains limited in regions where awareness is low or infrastructure is lacking. Several studies also emphasised the role of pharmacies as key touchpoints for ADR education and reporting. In Saudi Arabia and Australia, community pharmacies were identified as promising venues for integrating reporting processes into routine health interactions (Adopo et al., 2022; Kim et al., 2020). Across the included studies, notable differences emerged between LMICs and high-income countries (HICs) in patient awareness, engagement, and available reporting infrastructure.

In LMICs, awareness of ADR reporting systems was generally low, often hindered by insufficient health education, limited outreach campaigns, and underdeveloped reporting mechanisms that relied heavily on paper-based or in-person submissions (Adisa et al., 2019; Ata et al., 2021; Jha et al., 2017; Kadhim, 2015; Pahuja et al., 2014; Sirili et al., 2023). Digital literacy and internet access were also key barriers, reducing the adoption of mobile or online reporting tools (Basheera et al., 2022; Kassem et al., 2021; Muriithi et al., 2021; Sirili et al., 2023). In contrast, HICs such as the UK, Australia, and France demonstrated higher awareness and engagement, supported by structured public health campaigns, user-friendly national web portals, and integrated mobile applications (Adopo et al., 2022; Arnott et al., 2013; de Vries et al., 2021; Robertson & Newby, 2013). These findings highlight the importance of developing locally relevant pharmacovigilance measures, literacy levels, and technological capacity. Finally, various strategies were suggested to enhance patient participation in ADR reporting. Public education campaigns delivered via television, radio, and social media were recommended as primary tools to increase general awareness and knowledge (Getova et al., 2020; Jacobs et al., 2018; Ramia et al., 2021; Sirili et al., 2023). In addition, training healthcare providers to explain the importance of pharmacovigilance and assist patients in submitting reports proved effective in multiple settings.

Integrating reporting features into existing healthcare systems, such as electronic medical records and pharmacy management software, could help normalise reporting and reduce dependence on standalone platforms (Joaquim et al., 2023; Pahuja et al., 2014; Rehman et al., 2022). While meaningful efforts have been made to advance patient involvement in ADR reporting, challenges persist across multiple dimensions. Low health literacy, lack of awareness, poor access to digital infrastructure, and limited provider support continue to restrict patient engagement, particularly in low-resource settings. Nonetheless, the growing availability of digital reporting platforms, the influence of healthcare providers, and community-based education strategies represent substantial opportunities for improvement. By adopting a multifaceted and culturally responsive approach, healthcare systems can empower patients to play a more active role in medication safety through responsible ADR reporting.

## Conclusions

This scoping review provides a comprehensive synthesis of current evidence on patient awareness and involvement in ADR reporting across various countries, with particular emphasis on the facilitators, barriers, and strategic interventions that influence reporting behaviour. The findings demonstrate a persistent gap between patients’ awareness of ADRs and their actual involvement in formal pharmacovigilance activities. In many low and middle-income countries, patient knowledge of ADRs, their health implications, and available reporting mechanisms remains inadequate. This knowledge gap is compounded by infrastructural constraints, limited health literacy, and an absence of consistent feedback from reporting systems. On the other hand, high-income countries with well-established digital pharmacovigilance platforms and public health education campaigns tend to demonstrate higher levels of patient awareness and, in some cases, improved engagement. The introduction of user-friendly platforms such as mobile applications, web portals, and pharmacy-based systems has shown promising results in encouraging patient participation, especially when accompanied by healthcare provider support and community-based interventions. Nonetheless, despite the availability of digital tools in some regions, underutilisation persists due to a lack of awareness, poor usability, and sociocultural perceptions that reporting should be the sole responsibility of healthcare professionals. Therefore, a multifaceted approach that combines patient education, system simplification, and healthcare provider involvement is essential to bridge the gap between awareness and active participation in ADR reporting.

## Limitation

The review has several key limitations. First, it does not consider national disparities, reporting systems, or regulatory infrastructure**,** which may influence the reliability and generalizability of the data; for instance, findings from countries with robust pharmacovigilance systems may be more accurate than those from regions with limited reporting. Second, it equally weights very different study types**,** such as large national databases versus small interview studies with only 15 participants. Large-scale studies have greater statistical power and can detect rare events, whereas small studies provide limited insights, so treating them equivalently may bias conclusions. Third, by combining heterogeneous studies without accounting for methodological quality or sample size, the review introduces potential bias in its synthesis.

## Future recommendations

Based on the review findings, key recommendations to enhance patient ADR reporting include implementing nationwide, multi-channel public education campaigns through collaboration between health ministries, regulators, and community organisations. Moreover, reporting platforms and materials require simplification using user-centered design, plain language, local translations, and visual aids to improve accessibility. Furthermore, integration of ADR reporting into routine healthcare interactions, supported by trained frontline providers and embedded electronic prompts, is essential. Robust feedback mechanisms acknowledging patient contributions should be established within reporting systems. Governments must also play a role in promoting digital inclusion by expanding infrastructure and adapting successful mobile reporting applications such as the SFDA-ADR App (Saudi Arabia), the PVPI App (India), and the Yellow Card App (UK) for equitable access. Finally, further research is needed to evaluate culturally adapted interventions, digital literacy programmes, provider-patient communication models, and the specific needs of vulnerable subpopulations, especially the elderly, rural communities, and chronic disease patients, to develop the best pharmacovigilance strategies.

## Supplementary Material

Supplementary Material Quality Appraisal Tool.docx

## Data Availability

No new data were generated or analysed in this study. All data are available in the published articles included in the review.
