# Supplementary material for: Strengthening pharmacovigilance through patient reporting: a scoping review of awareness, barriers, and facilitators
Source: J Pharm Policy Pract. 2026 Apr 13;19(1):2651405. doi: 10.1080/20523211.2026.2651405 (PMC13072706; doi:10.1080/20523211.2026.2651405)
Supplement: Supplementary Material Quality Appraisal Tool.docx [file JPPP_A_2651405_SM2162.docx]

**Supplementary Material 3: Quality Appraisal for Studies Included**

**Figure 1** Methodological quality assessment of included studies using the Mixed Methods Appraisal Tool (MMAT) 2018


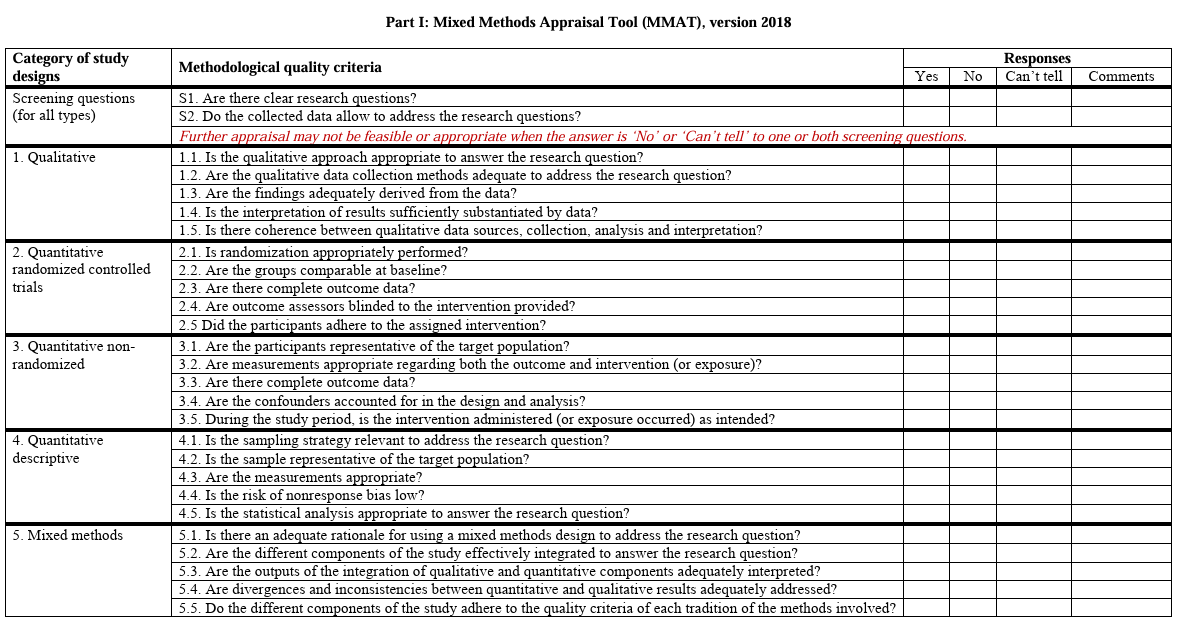


*Note.* The Mixed Methods Appraisal Tool (MMAT), version 2018, was used to assess the methodological quality of the included studies. The tool evaluates five core criteria for each study type (qualitative, quantitative descriptive, quantitative non-randomised, quantitative randomised controlled, and mixed methods), enabling consistent appraisal across diverse designs.

**Table 1** Methodological quality appraisal of included studies using the Mixed Methods Appraisal Tool (MMAT) 2018

| **Author (Year), Country** | **Screening Questions** | | **Qualitative** | | | | | **Quantitative descriptive** | | | | | **Mixed methods** | | | | | **Quality** |
| --- | --- | --- | --- | --- | --- | --- | --- | --- | --- | --- | --- | --- | --- | --- | --- | --- | --- | --- |
|  | **S1** | **S2** | **1.1** | **1.2** | **1.3** | **1.4** | **1.5** | **4.1** | **4.2** | **4.3** | **4.4** | **4.5** | **5.1** | **5.2** | **5.3** | **5.4** | **5.5** | **High (4-5)**  **Moderate (2-3)**  **Low (0-1)** |
| Aamir et al. (2024)  India [1] | Y | Y |  |  |  |  |  | Y | Y | Y | Y | Y |  |  |  |  |  | High |
| Adisa et al. (2019)  Nigeria [2] | Y | Y |  |  |  |  |  | Y | ? | Y | N | Y |  |  |  |  |  | Moderate |
| Adopo et al. (2022)  France [3] | Y | Y |  |  |  |  |  | Y | Y | Y | Y | Y |  |  |  |  |  | High |
| Aldeyab et al. (2016)  UK [4] | Y | Y |  |  |  |  |  | Y | Y | Y | ? | Y |  |  |  |  |  | High |
| Almubark et al. (2020)  Saudi Arabia [5] | Y | Y |  |  |  |  |  | Y | N | Y | Y | Y |  |  |  |  |  | High |
| Al Dweik et al. (2020)  Canada [6] | Y | Y | Y | Y | Y | Y | Y |  |  |  |  |  |  |  |  |  |  | High |
| Al Dweik et al. (2020)  Canada [7] | Y | Y |  |  |  |  |  | ? | ? | Y | N | Y |  |  |  |  |  | Moderate |
| Appiah et al. (2019)  Ghana [8] | Y | Y | Y | Y | Y | Y | Y |  |  |  |  |  |  |  |  |  |  | High |
| Arnott et al. (2013)  UK [9] | Y | Y | Y | Y | Y | Y | Y |  |  |  |  |  |  |  |  |  |  | High |
| Ata et al. (2021)  Bangladesh [10] | Y | Y |  |  |  |  |  | Y | Y | Y | Y | Y |  |  |  |  |  | High |
| Aydınkarahaliloğlu et al. (2018)  Turkey [11] | Y | Y |  |  |  |  |  | Y | Y | Y | Y | Y |  |  |  |  |  | High |
| Basheera et al. (2022)  India [12] | Y | Y |  |  |  |  |  | Y | ? | Y | Y | Y |  |  |  |  |  | High |
| Bhoombla et al. (2020)  UK [13] | Y | Y |  |  |  |  |  | Y | Y | Y | ? | Y |  |  |  |  |  | High |
| Dedefo et al. (2024)  Australia [14] | Y | Y |  |  |  |  |  | Y | Y | Y | Y | Y |  |  |  |  |  | High |
| de Vries et al. (2021)  Croatia, Netherlands, UK [15] | Y | Y |  |  |  |  |  | Y | Y | Y | Y | Y |  |  |  |  |  | High |
| El-Dahiyat et al. (2023)  Jordan [16] | Y | Y |  |  |  |  |  | Y | Y | Y | Y | Y |  |  |  |  |  | High |
| Getove et al. (2020)  Bulgaria [17] | Y | Y |  |  |  |  |  | Y | N | Y | Y | Y |  |  |  |  |  | High |
| Jacobs et al. (2018)  Ghana [18] | Y | Y |  |  |  |  |  |  |  |  |  |  | Y | Y | Y | Y | Y | High |
| Jha et al. (2017)  Nepal [19] | Y | Y |  |  |  |  |  | Y | Y | Y | ? | Y |  |  |  |  |  | High |
| Joaquim et al. (2023)  Portugal [20] |  |  |  |  |  |  |  | Y | ? | Y | ? | Y |  |  |  |  |  | Moderate |
| Julian et al. (2018)  Brazil [21] | Y | Y |  |  |  |  |  | Y | Y | Y | Y | Y |  |  |  |  |  | High |
| Kadhim (2015)  Iraq [22] | Y | Y |  |  |  |  |  | Y | ? | Y | N | Y |  |  |  |  |  | Moderate |
| Kassem et al. (2021)  Saudi Arabia [23] | Y | Y | Y | Y | Y | Y | Y |  |  |  |  |  |  |  |  |  |  | High |
| Kim et al. (2020)  Korea [24] | Y | Y |  |  |  |  |  | Y | Y | Y | Y | Y |  |  |  |  |  | High |
| Matlala et al. (2023)  South Africa [25] | Y | Y |  |  |  |  |  | Y | Y | Y | Y | Y |  |  |  |  |  | High |
| Matos et al. (2015)  Portugal [26] | Y | Y |  |  |  |  |  | Y | Y | Y | ? | Y |  |  |  |  |  | High |
| Muriithi et al. (2021)  Kenya [27] | Y | Y |  |  |  |  |  | Y | Y | Y | Y | Y |  |  |  |  |  | High |
| Noda et al. (2020)  Japan [28] | Y | Y |  |  |  |  |  | Y | Y | Y | Y | Y |  |  |  |  |  | High |
| Nyagah & Muriithi (2021)  Kenya [29] | Y | Y |  |  |  |  |  |  |  |  |  |  | Y | Y | Y | ? | Y | High |
| Pahuja et al. (2014)  India [30] | Y | Y |  |  |  |  |  | Y | N | Y | Y | Y |  |  |  |  |  | High |
| Patel et al. (2019)  India [31] | Y | Y |  |  |  |  |  | Y | Y | Y | Y | Y |  |  |  |  |  | High |
| Ramia et al. (2021)  Lebanon [32] | Y | Y |  |  |  |  |  | Y | Y | Y | Y | Y |  |  |  |  |  | High |
| Rehman et al. (2022)  Pakistan [33] | Y | Y |  |  |  |  |  | Y | Y | Y | Y | Y |  |  |  |  |  | High |
| Robertson et al. (2013)  Australia [34] | Y | Y |  |  |  |  |  | Y | ? | Y | N | Y |  |  |  |  |  | Moderate |
| Sabblah et al. (2017)  Ghana [35] | Y | Y | Y | Y | Y | Y | Y |  |  |  |  |  |  |  |  |  |  | High |
| Sirili et al. (2023)  Tanzania [36] | Y | Y |  |  |  |  |  | Y | Y | Y | Y | Y |  |  |  |  |  | High |
| Srisuriyachanchai et al. (2023)  Thailand [37] | Y | Y |  |  |  |  |  | Y | Y | Y | Y | Y |  |  |  |  |  | High |
| Staniszewska et al. (2016)  Poland [38] | Y | Y |  |  |  |  |  | Y | Y | Y | ? | Y |  |  |  |  |  | High |
| Tejus et al. (2020)  India [39] | Y | Y |  |  |  |  |  | Y | Y | Y | Y | Y |  |  |  |  |  | High |
| Thadani et al. (2019)  India [40] | Y | Y |  |  |  |  |  | Y | Y | Y | Y | Y |  |  |  |  |  | High |
| Van Hunsel et al. (2017)  Netherlands [41] | Y | Y |  |  |  |  |  | Y | ? | Y | N | Y |  |  |  |  |  | Moderate |
| Wang et al. (2023)  China [42] | Y | Y | Y | Y | Y | Y | Y |  |  |  |  |  |  |  |  |  |  | High |

**Note:** *Y = Yes; N = No; ? = Unclear.*

**REFERENCES**

1. Kushwaha, V., et al., *Questionnaire Based Survey to Assess Awareness on Drug Safety, ADR and ADR Reporting on Patients Attending OPD/IPD in a Tertiary Care Hospital, Kanpur During National Pharmacovigilance Week.* International Journal of Pharmaceutical Sciences Review and Research, 2024. **84**: p. 130-135.

2. Adisa, R., O.R. Adeniyi, and T.O. Fakeye, *Knowledge, awareness, perception and reporting of experienced adverse drug reactions among outpatients in Nigeria.* International journal of clinical pharmacy, 2019. **41**: p. 1062-1073.

3. Adopo, D., et al., *Patient engagement in Pharmacovigilance: determinants and evolution of reporting from 2011 to 2020 in France.* 2022.

4. Aldeyab, M.A., et al., *Assessment of the impact of the Scottish public health campaign on patient reporting of adverse drug reactions.* Drugs & Therapy Perspectives, 2016. **32**: p. 209-218.

5. Almubark, R.A., et al., *National cross-sectional study of community-based adverse drug reactions in Saudi Arabia.* Drugs-real world outcomes, 2020. **7**(2): p. 161-170.

6. Al Dweik, R., et al., *Patients' experiences on adverse drug reactions reporting: a qualitative study.* Eur J Clin Pharmacol, 2020. **76**(12): p. 1723-1730.

7. Al Dweik, R., et al., *Adverse drug reaction reporting in Canada: consumer versus physician reports.* Drugs & Therapy Perspectives, 2020. **36**: p. 469-475.

8. Appiah, B., et al., *Factors that influence the intention to use mobile phone caller tunes for patient reporting of adverse drug reactions: a qualitative study.* Therapeutic Advances in Drug Safety, 2019. **10**: p. 2042098619871190.

9. Arnott, J., et al., *What can we learn from parents about enhancing participation in pharmacovigilance?* British journal of clinical pharmacology, 2013. **75**(4): p. 1109-1117.

10. Ata, M., et al., *A Cross Sectional Study on Awareness of Consumers to Adverse Drug Reaction Reporting Related Aspects in a Tertiary Medical College Hospital.* Journal of Army Medical College Chattogram, 2021. **4**(1): p. 21-24.

11. Aydınkarahaliloğlu, N.D., et al., *Spontaneous reporting of adverse drug reactions by consumers in comparison with healthcare professionals in Turkey from 2014 to 2016.* Pharmaceutical Medicine, 2018. **32**: p. 353-364.

12. Basheera, V., et al., *A Step to Improve Pharmacovigilance System: A Cross Sectional Study among Outpatients Visiting Multi Super Speciality Hospital in Kerala Established with ADR Monitoring Centre.* Indian Journal of Pharmacy Practice, 2022. **15**(4).

13. Bhoombla, N., et al., *Pharmacovigilance reports received from children and young people, and development of information to aid future reporting from this age group.* Pediatric Drugs, 2020. **22**: p. 335-341.

14. Dedefo, M.G., et al., *Consumers’ knowledge and experiences of adverse drug reaction reporting in Australia: a national survey.* European Journal of Clinical Pharmacology, 2024. **80**(10): p. 1543-1554.

15. de Vries, S.T., et al., *Motives to report adverse drug reactions to the national agency: a survey study among healthcare professionals and patients in Croatia, The Netherlands, and the UK.* Drug Safety, 2021. **44**: p. 1073-1083.

16. El-Dahiyat, F., et al., *Jordanians' knowledge, attitude and practice regarding adverse drug reactions reporting.* Saudi Pharmaceutical Journal, 2023. **31**(7): p. 1197-1201.

17. Getova, V., S. Gueorgiev, and I. Getov, *Evaluation of Patients’ Knowledge on the Black Triangle Symbol and Meaning–Bulgarian Perspective.* Enliven: Pharmacovigilance and Drug Safety, 2020. **6**(3): p. 019.

18. Jacobs, T.G., et al., *The contribution of Ghanaian patients to the reporting of adverse drug reactions: a quantitative and qualitative study.* BMC Public Health, 2018. **18**: p. 1-11.

19. Jha, N., et al., *Knowledge, attitude and practice regarding Pharmacovigilance and consumer Pharmacovigilance among consumers at Lalitpur District, Nepal.* 2017.

20. Joaquim, J., C. Matos, and R. Mateos-Campos, *Assessment of risk perception of patients concerning adverse drug reactions.* Current Issues in Pharmacy and Medical Sciences, 2023. **36**: p. 103-107.

21. Julian, G.S., et al., *Pharmacovigilance knowledge in Brazil: perception of participants of oncology patient advocacy group on adverse events reporting.* Brazilian Journal of Oncology, 2018. **14**(48): p. 1-11.

22. Kadhim, K.A., *Self-reporting of adverse drug reactions in Iraqi hospitals: patient’s perspectives.* Pharmacology & Pharmacy, 2015. **6**(12): p. 566-572.

23. Kassem, L.M., et al., *Understanding patient needs regarding adverse drug reaction reporting smartphone applications: a qualitative insight from Saudi Arabia.* International Journal of Environmental Research and Public Health, 2021. **18**(8): p. 3862.

24. Kim, S., et al., *A cross-sectional survey of knowledge, attitude, and willingness to engage in spontaneous reporting of adverse drug reactions by Korean consumers.* BMC Public Health, 2020. **20**: p. 1-11.

25. Matlala, M., M. Lubbe, and H. Steyn, *Profile of adverse drug reaction reports in South Africa: An analysis of VigiBase for the year 2017.* South African Medical Journal, 2023. **113**(6): p. 1205-1212.

26. Matos, C., F. Van Hunsel, and J. Joaquim, *Are consumers ready to take part in the Pharmacovigilance System?—A Portuguese preliminary study concerning ADR reporting.* European journal of clinical pharmacology, 2015. **71**: p. 883-890.

27. Muriithi, D., et al., *Factors influencing adverse drug reaction reporting among patients in selected hospitals within Kirinyaga County, Kenya.* 2021.

28. Noda, A., et al., *Characteristics of pediatric adverse drug reaction reports in the Japanese Adverse Drug Event Report Database.* BMC Pharmacology and Toxicology, 2020. **21**: p. 1-10.

29. Nyagah, D.M., *Factors Influencing Adverse Drug Reaction Reporting among Patients and Healthcare Providers in Selected Hospitals in Kirinyaga County, Kenya*. 2021, JKUAT-COHES.

30. Pahuja, R., et al., *Awareness on adverse drug reaction reporting system in India: a consumer survey.* American Journal of Phytomedicine and Clinical Therapeutics, 2014. **2**(12): p. 1361-1369.

31. Patel, J.J., et al., *Knowledge, attitude and practice among consumers about adverse drug reaction reporting.* International Journal of Basic & Clinical Pharmacology, 2019. **8**(8): p. 1776.

32. Ramia, E., et al., *A population-based study of self-reported adverse drug events among Lebanese outpatients.* Scientific Reports, 2021. **11**(1): p. 7921.

33. Rehman, A.U., et al., *Patients’ perception of the pharmacovigilance system: A pre-diagnostic and post-interventional cross-sectional survey.* Frontiers in Pharmacology, 2022. **13**: p. 936124.

34. Robertson, J. and D.A. Newby, *Low awareness of adverse drug reaction reporting systems: a consumer survey.* Medical Journal of Australia, 2013. **199**(10): p. 684-686.

35. Sabblah, G.T., et al., *Patients’ perspectives on adverse drug reaction reporting in a developing country: a case study from Ghana.* Drug safety, 2017. **40**: p. 911-921.

36. Sirili, N., et al., *Awareness, Actions, and Predictors of Actions on Adverse Drug Reaction Reporting among Patients Attending a Referral Hospital in Southern Highland Tanzania.* Advances in Pharmacological and Pharmaceutical Sciences, 2023. **2023**(1): p. 7761649.

37. Srisuriyachanchai, W., et al., *Severity and management of adverse drug reactions reported by patients and healthcare professionals: a cross-sectional survey.* International Journal of Environmental Research and Public Health, 2023. **20**(4): p. 3725.

38. Staniszewska, A., et al., *Patient knowledge on reporting adverse drug reactions in Poland.* Patient preference and adherence, 2016: p. 47-53.

39. Tejus, A., et al., *An observational study to assess the possibility of patient participation in implementing pharmacovigilance in a busy tertiary care hospital.* medical journal armed forces india, 2020. **76**(4): p. 425-429.

40. Thadani, A., et al., *Evaluation of knowledge and awareness of adverse drug reaction reporting among patients visiting a tertiary care hospital in northern India.* Asian Journal of Pharmacy and Pharmacology, 2019. **5**(2): p. 310-315.

41. van Hunsel, F., S. De Waal, and L. Härmark, *The contribution of direct patient reported ADRs to drug safety signals in the Netherlands from 2010 to 2015.* Pharmacoepidemiology and Drug Safety, 2017. **26**(8): p. 977-983.

42. Wang, D., et al., *Views on suspected adverse drug events in older adults with chronic conditions: A qualitative study.* Patient preference and adherence, 2023: p. 2051-2061.
